# Itch in Hymenoptera Sting Reactions

**DOI:** 10.3389/falgy.2021.727776

**Published:** 2021-08-20

**Authors:** Urban Cerpes, Maria-Lisa Repelnig, Franz J. Legat

**Affiliations:** Department of Dermatology and Venerology, Medical University of Graz, Graz, Austria

**Keywords:** insect sting, Hymenoptera venom, pathophysiology, itch, venom activity

## Abstract

Insect stings and the resulting itch are a ubiquitous problem. Stings by members of the insect order Hymenoptera, which includes sawflies, wasps, bees and ants, and especially by bees and wasps are extremely common, with 56–94% of the population being stung at least once in their lifetime. The complex process of venom activity and inflammation causes local reactions with pain and pruritus, sometimes anaphylactic reactions and more seldomly, as in case of numerous stings, systemic intoxication. We reviewed the literature regarding itch experienced after Hymenoptera stings, but found no study that placed a specific focus on this topic. Hymenoptera venoms are composed of many biologically active substances, including peptide toxins and proteinaceous toxins. Peptide toxins from bee venom cause cell lysis and ion channel modulation in the peripheral and central nervous systems, while toxins from wasp venom induce mast cell degranulation and chemotaxis of polymorphonuclear leukocytes in the skin. The proteinaceous toxins cause a disruption of the cell membranes and necrotic cell death, degradation of hyaluronan (an extracellular matrix glycosaminoglycan), increased vascular permeability, hemolysis, as well as activated platelet aggregation. Mediators which could be directly involved in the venom-induced pruritus include histamine and tryptase released from mast cells, interleukin-4 and interleukin-13 from Th2 lymphocytes, as well as leukotriene C4. We postulate that a pruriceptive itch is induced due to the pharmacological properties of Hymenoptera venoms.

## Introduction

Hymenoptera are a large order in the class of insects, which include sawflies (Symphyta), bees (Apoidae), wasps (Vespidae) and ants (Myrmicidiae). Hymenoptera stings are very common, with 56–94% of the population being stung at least once in their lifetime (1). Their venoms are natural weapons used for defense and/or predation, containing a complex mix of bioactive molecules, proteinaceous compounds and peptides (2). The envenomation process causes local inflammatory reactions accompanied by pain and pruritus, and more seldomly systemic intoxication after numerous stings (3, 4). The majority of medical important stings are caused by social Hymenoptera. In the clinic, anaphylaxis is the most important consequence observed after Hymenoptera stings, which are the most common triggers for anaphylaxis in adulthood in Europe (5, 6). However, Hymenoptera venoms are of interest due to their potential to cause anaphylaxis and inflammation, but also because they contain highly active biological compounds and are of interest as anti-microbial, anti-tumoral and anti-inflammatory therapeutics (7–9).

Pruritus is an important and highly frequent sensation, which is experienced by any human being from time to time. Pruritus causes the desire to scratch, which is believed to assist in the removal of irritants, and, in the case of insect stings, to remove the inserted stinger or the whole insect from the skin. In recent years, many advances have been made that have improved our understanding of the pathogenesis of pruritus. In many cases, interactions have been found between inflammatory mediators and the peripheral and central nervous systems (10, 11). Prurioceptic neurons are currently classified as either histamine-sensitive or histamine-insensitive neurons. Histamine, a biogenic amine, is capable of causing local immune cell activation and chemotaxis as well as regulation (12–14). This amine induces itch by binding to histamine receptors (H1R and H4R subtypes) located on histamine-sensitive sensory neurons, which have cell bodies located in the dorsal root ganglia (15). Non-histaminergic itch can also be mediated via various pathways such as protease-activated receptors, mas-related G protein-coupled receptors (MRGPR), or cytokine receptors (16, 17). Cytokine receptors involved in non-histaminergic itch can be activated by several cytokines such as IL-4, IL-13, IL-31, IL-33, as well as thymic stromal lymphopoietin (TSLP) (18–22). In both histaminergic and non-histaminergic pathways, the transduction of the itch signal involves the activation of ion channels known as transient receptor potential channels (TRP), which include the important TRPV (vanilloid) and TRPA (ankyrin) subfamilies (23, 24). The stimulus is transmitted mainly via C-type neurons and to a lesser extent through Aδ-neurons to the dorsal root ganglia, and then on to the dorsal horn of the spinal cord. The itch signal is then transmitted by inter-neurons to nerve fibers; the signal then crosses to the contralateral side, ascends through the spinothalamic tract to the thalamus and continues on to various brain regions (17, 25).

We conducted a literature review with the online search engine PubMed, which provides access to ca. 32 million citations from the MEDLINE bibliographic database, life science journals and online books, to identify publications on itch experienced after Hymenoptera stings. No study that placed a primary focus on this topic was found. Based on our own experience conducting sting provocation trials with live bees and wasps as a therapy control as part of an ongoing venom immunotherapy program, patients initially complain of pain and report experiencing pruritus within 20–30 min after being stung.

## Components of Hymenoptera Venoms

In this section, we present some of the main components of Hymenoptera venoms which might be implicated in the pathophysiology of itch. Many other components are known, and an excellent summary of these is provided in the review by dos Santos-Pinto et al. (26). The venoms of the species of the respective families do not contain entirely homologous proteins and peptides and, in the case of ants, have been poorly studied.

### Bee Venom Components

#### Melittin

The main component of bee venom is melittin, which accounts for 40–60% of the dry venom weight. Melittin is a basic 26-amino-acid polypeptide (https://pubchem.ncbi.nlm.nih.gov/compound/16133648) which has cytolytic properties, causes muscle contractions, triggers the release of histamine and disrupts surface tension. It is considered to be one of the main pain-causing agents in bee venom (27). Melittin causes activation of transient receptor potential channel vanilloid 1 (TRPV1), a non-selective cation channel on peripheral sensory neurons (28). This receptor is implicated in nociception and the sensation of itch and is stimulated by histamine (29), but can also be stimulated by other important itch-inducing cytokines such as IL-31 and IL-4 (19). The receptor can be activated by its ligands, which are produced through the cyclooxygenase (COXs), lipoxygenase (LOXs) and phospholipase 2 (PLA 2) pathways (27). Another way to induce pruritus by melittin could be via the release of serotonin due to pore formation and mast cell degranulation (27). Melittin also increases transcriptional regulation of voltage-gated sodium channels on neurons associated with itch (17, 30). In addition, this polypeptide also activates phospholipase A2 (31).

#### Phospholipase A2

Phospholipase A2 (PLA) is a 16–18 kDa protein, which accounts for ~12% of dry bee venom weight (32). It hydrolyses the sn-2 ester bond of glycerophospholipids in biological membranes to release free fatty acids and lysophospholipids (33). It also displays neurotoxic properties by binding to the N-type PLA2 receptor on neurons (34). When injected into rodents' skin, it has been shown that it induces the IgE-independent release of mediators from mast cells, such as pruritogenic IL-4 (35). Furthermore, it can induce a Th2 type cell response as well as the release of IL-33 (36). In a mouse model study, IL-33 and its receptor ST2 were implicated in pruritus due to poison ivy contact (20). Human PLA2 is also involved in reactions to lipoxygenase metabolites in the intracellular cascade of nociceptive and histamine-dependent itch signaling (37).

#### Hyaluronidase

Hyaluronidase is a constituent of the venoms from many Hymenoptera species. It is a 45-kDa protein that degrades hyaluronic acid, an abundant glycosaminoglycan present in the extracellular matrix (38). It has been shown to increase the absorption, penetration of venoms and consequently their activity (39), which might also enhance itch.

### Vespid Venom Components

#### Mastoparans

Mastoparans are the most abundant proteins in wasp venoms, consisting of 12–14 amino acids (40). These proteins can cause cell lysis and IgE-independent mast cell degranulation, triggering the release of proinflammatory pruritogenic substances such as histamine, tryptase, leukotriene C4 (LTC 4), IL-4, IL-31, and IL-33 (26, 41, 42). Tryptase has been shown to activate protease-activated receptor 2 (PAR-2), which can cause itch flares in patients with atopic dermatitis. LTC 4 can mediate itch through the cysteinyl leukotriene receptor 2 (CysLTR2) on sensory nerves (43, 44).

#### Phospholipase 1

Phospholipase 1 (PLA1) is a non-glycosylated protein which is ~20–30% homologous to the human lipase (45). It causes the disruption of phospholipids in the cell membrane and the formation of pores, leading to cell lysis, hemolysis and the activation of platelet aggregation (45–47). Researchers have demonstrated that PLA1 promotes the release of prostaglandin E2 (PGE2) from macrophages, which, in turn, induces Th2 inflammation with the release of Th2 type cytokines, such as the pruritogenic IL-4 (48).

#### Kinin-Related Peptides

Kinin-related peptides are polypeptides containing 9–18 amino acids, which are structurally similar to human pain-stimulating peptide bradykinin (49). Bradykinin plays an important role in inflammation in that it increases blood flow and vascular permeability. Via arachidonic acid metabolites and protein kinase C, it also causes pain by activating TRPV1, which is also implicated in itch (50). Furthermore, bradykinin can stimulate neurosensory nerves by activating bradykinin B2-receptors, eventually inducing neurogenic inflammation with the release of CGRP (calcitonin gene-related peptide) and substance P, which again can directly and indirectly induce itch (51, 52). In addition, researchers reported that bradykinin causes itch when administered to human skin through iontophoresis (53).

### Ant Venom

Due to lack of data on this topic, we do not focus on the specific proteins and components in ant venom but briefly review the known components.

#### Ant Venom Alkaloids

Venom alkaloids seem to be a unique feature of ants among Hymenopterans. Alkaloids in ants have been reported from an increasing number of different ant groups. Their structures are considerably diverse; however, alkaloids present in the venom from ants of the same species group tend to share the same basic structure. The chemistry and physiological effects of venom alkaloids have been most comprehensively studied in the fire ants. The alkaloids in fire ant venoms are mainly hydrophobic piperidines called solenopsins, while piperideines appear in much lower amounts. Solenopsins were shown to trigger histamine production in mastocytes, activating platelets and neutrophils, causing a blockade of the neuromuscular junction, inhibiting ATP-dependent sodium-potassium pumps and inhibiting neuronal nitric oxide synthase (54). While histamine release and mast cell degranulation are well-described mechanisms of itch, inhibiting neuronal nitric oxide synthase was shown to reduce itch in chloroquine-induced scratching (55). While platelet activation may lead to serotonin release and, therefore, induce itch, the study findings also indicate that the presence of neutrophils may lead to an upregulation of itch (17, 56).

#### Ant Venom Proteins

The exact immunological response to each venom component has not been empirically identified. However, a study exploring the allergic response mediated by fire ant venom proteins gives some insight into the physiological effects of ant venom proteins.

This study showed that the injection of ant venom proteins in previously sensitized mice led to an increase in eosinophils (57).

Eosinophils are a major cellular source of the highly pruritogenic cytokine IL-31 in bullous pemphigoid (58). Since ant venom proteins have shown to promote eosinophil recruitment, this may lead to an increased expression of IL-31 and, therefore, induce itch.

Ant venom protein exposure can also lead to dendritic cell activation and elevated IL-4 (57).

#### Ant Venom Peptides

To date at least 75 venom peptides from 11 ant species have been completely sequenced. These peptides can be grouped into three categories: cytolytic, neurotoxic and uncharacterized peptides. Most proteomic studies on ant venoms have shown the prevalence of small, linear peptides. Most of these small peptides possess cytolytic properties and also display hemolytic and insecticidal properties. Ponericins, dinoponeratoxins, pilosulins, and bicarinalins are included in this group of cytolytic peptides. It is believed that the cytolytic peptides identified in ant venoms are multifunctional; they act as membrane-disrupting agents, facilitate the diffusion of other neurotoxins and exhibit antimicrobial activity.

The neurotoxic properties of ant venom peptides have rarely been reported, and only a few neurotoxic peptides have been characterized (54). Poneratoxin modulates voltage-gated sodium (Nav) channels, which are also associated with itch (17). Ectatomin and poneritoxin are L-type voltage-gated calcium channel blockers, and ectatomin is additionally a pore-forming peptide (26). The pore forming process may lead to serotonin release, which can also be an inductor of itch. The complete sequence, structure and biological functions are still unknown for uncharacterized peptides, such as myrmexins and ICK-like peptides (26).

## Potential Mechanisms for Itch From Hymenoptera Stings

**Figure 1:**
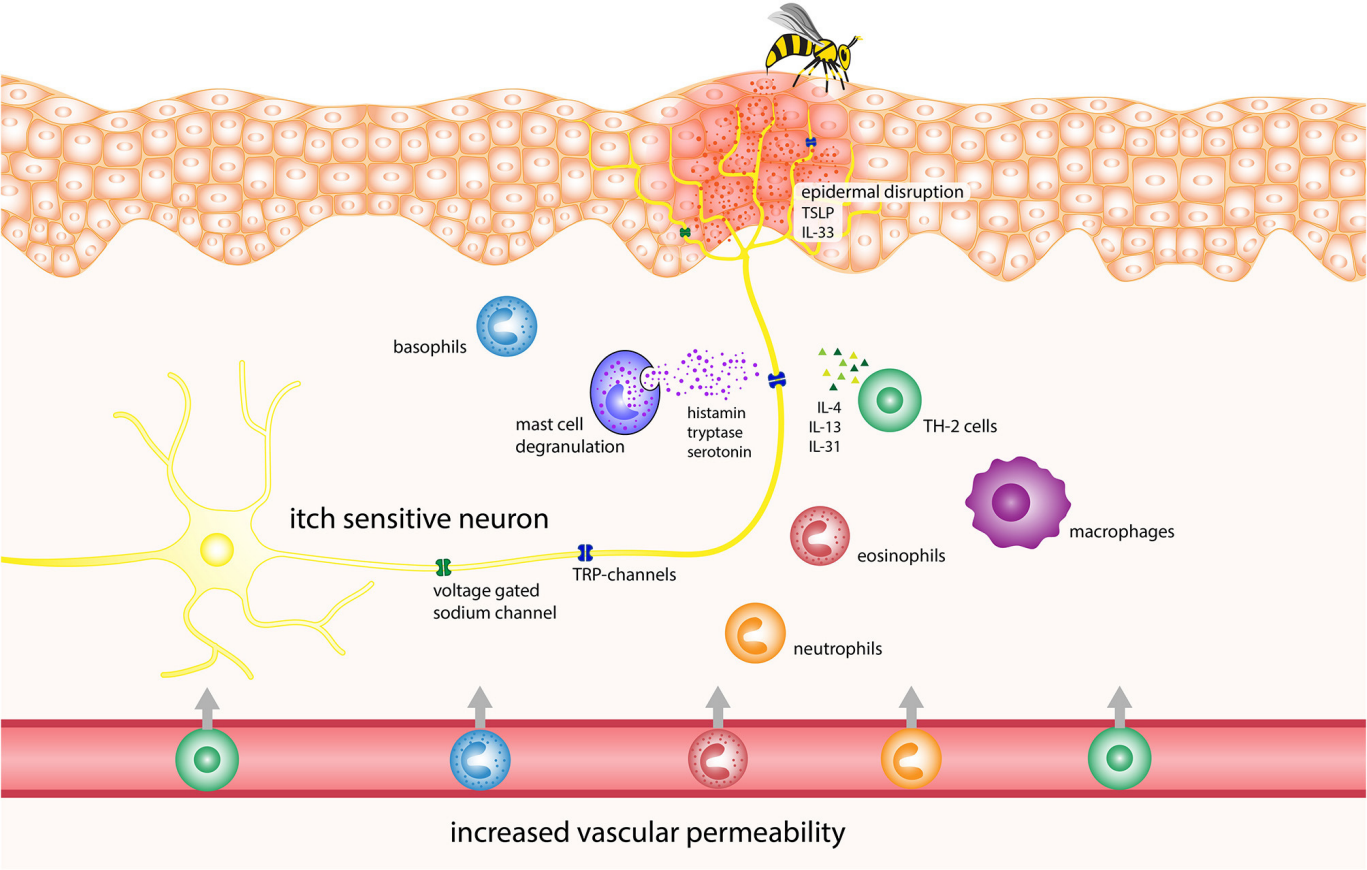
Potential pathophysiology of itch induction in Hymenoptera stings. IL, interleukin; TRP, transient receptor potential channels.

Since Hymenoptera venoms contain an abundant array of bioactive components, the separate examination of each of these would represent an overly simplistic und inadequate approach. Thus, in the next section, we discuss possible pathophysiological mechanisms of itch due to the epidermal disruption and the venom, placing a focus on immune system interplay. Table 1 lists the Hymenoptera venom components, with potential of itch induction and Figure 1 displays the potential pathophysiology of itch induction in Hymenoptera stings.

**Table 1:** Components of Hymenoptera venoms with their potential mechanism(s) of itch induction.

**Family**	**Venom component**	**Pathophysiology**	**Potential mechanism(s) of itch induction**
Apoidea	Melittin	Cytolytic properties, Release of histamine and disruption of surface tension	Activation of TRPV1 on sensory neurons, release of serotonin due to pore formation
Apoidea	PLA2	Neurotoxicity via binding N-Type PLA2 receptors on neurons, hydrolysation of glycerophospholipids	IgE independent degranulation of mast cells, induce Th2 Type immune response, human PLA2 is involved in the intracellular histamine dependent itch signaling
Apoidea, Vespidae	Hyaluronidase	Degradation of hyaluronic acid	Increases the penetration and activity of the venom
Vespidae	Mastoparans	Cell lysis and IgE-independent mast cell degranulation	Release of pruritogens from mast cells: Histamine, tryptase, LTC 4, IL-4, IL-31, and IL-33
Vespidae	PLA1	Disruption of phospholipids in the cell membrane and the formation of pores, leading to cell lysis, hemolysis, platelet aggregation	Induction of Th2 driven response over the release of PGE2 from mast cells
Vespidae	Kinin-related peptides	Induction of inflammation, increases blood flow and vascular permeability	Induction of neurogenic inflammation with the release of CGRP and substance P
Myrmicidiae	Solenopsins	Inducing histamine production in mastocytes, activating platelets and neutrophils, blocking the neuromuscular junction, mast cell degranulation	Histamine release, mast cell degranulation, inhibiting neuronal nitric oxide synthase

*TRPV1, transient receptor potential channel vanilloid 1; PLA1, phospholipase A1; PLA2, phospholipase A2; IL, interleukin; LTC4, leukotriene C4; PGE2, prostaglandin E2; CGRP, calcitonin gene-related peptide*.

### Epidermal Disruption

The initial stimulus for pruritus after Hymenoptera stings is believed to be due to damage caused to the epidermal barrier by the sting. This damage activates the immune cells, triggers the degranulation of mast cells and attracts circulating leukocytes (59, 60), resulting in the eventual release of TSLP, prostaglandin, histamine, serotonin, endothelin, nerve growth factor, as well as cytokines and chemokines (61). TSLP can trigger itch via direct neuronal activation as well as by initiating a Th2-type inflammation process, which involves the release of pruritogenic IL-4 and IL-13 (62, 63). Although these cytokines are primarily associated with chronic itch, they can also activate sensory neurons and increase their responsiveness to other itch mediators (19). Furthermore, the venom itself contains biogenic amines such as histamine and serotonin. Studies have shown that the latter can cause itch in humans and scratching in mice when injected into the skin (64, 65).

### Immune Response to Venom

The immune response to venoms is mediated through mast cells, cell lysis and proinflammatory venom components, as described earlier. In addition, researchers have shown in animal models that honey bee venom attracts neutrophils and causes the rapid release of IL-1b, TNF-α and especially IL-6, as well as tachykinins such as substance P and neurokinin A (66, 67). Tachykinins released from sensory nerve fibers promote inflammation and cause edema via the neurokinin (NK)-1, NK-2 and NK-3 receptors. NK-1 receptors are found on mast cells, as well as other cells, where their activation can cause mast cell degranulation (51, 68). Furthermore, TNF-α has been implicated in acute itch in mice by sensitizing afferent neurons to other pruritogens (69). Higher serum levels of IL-6 have also been shown to correlate with higher itch levels in patients with prurigo nodularis (70), although the exact mechanism of IL-6-induced itch is not known.

### IgE-Mediated Itch

Asymptomatic IgE-sensitization to Hymenoptera venoms is common, with 27.1–40.7% of the general population having detectable levels of specific IgE against them, although systemic sting reactions rarely occur in these people (71–73). Due to this fact, itch could be elicited via IgE-crosslinking of FcεRI receptors mediating mast cell degranulation and the eventual release of pruritogenic mediators that were mentioned previously in the section describing mastoparans (42). Another way that IgE might cause itch is via the basophil-neuronal axis, which has been implicated in itch during flare-ups of acute atopic dermatitis. When allergen-IgE binds to basophils, it causes the release of LTC4; this substance then binds to the LTC4 receptor CysLTR2 on afferent sensory neurons and causes pruritus via the activation of either TRPV1 or TRPA1 (74).

## Conclusions

Hymenoptera venoms contain a diversity of biochemical substances and have complex biochemical properties. Due to the absence of comprehensive studies on the pruritic activity of Hymenoptera venoms, we can only speculate on the mechanisms of itch experienced after Hymenoptera stings. We suggest that the pruritus observed after Hymenoptera stings is induced by a combination of the epidermal disruption caused by the sting, the direct effects of the venom on afferent sensory neurons and pruritogenic, Th2-driven immune responses. Cells that play key roles in the skin during these processes include the mast cells, basophils, Th2 lymphocytes and keratinocytes. These cells release a wide range of bioactive compounds in response, such as histamine, serotonin, IL-4, IL-13, IL-31, and LTC4. All of these have been implicated in the direct or indirect induction of itch and mainly depend on the activity of TRPV1 and TRPA1 channels in the peripheral sensory neurons. The insufficient knowledge about the pathophysiology of pruritus after Hymenoptera stings highlights the necessity to conduct further studies on this topic, to address this knowledge gap and collect further evidence on this commonly-occurring event.

## Author Contributions

UC, M-LR, and FJL reviewed literature, interpreted the data, and authored and revised the manuscript. UC and M-LR were the main authors of the manuscript. FJL supervised and critically discussed the manuscript with the other authors. All gave their final approval.

## Conflict of Interest

The authors declare that the research was conducted in the absence of any commercial or financial relationships that could be construed as a potential conflict of interest.

## Publisher's Note

All claims expressed in this article are solely those of the authors and do not necessarily represent those of their affiliated organizations, or those of the publisher, the editors and the reviewers. Any product that may be evaluated in this article, or claim that may be made by its manufacturer, is not guaranteed or endorsed by the publisher.
